# Small T Oncoprotein of Merkel Cell Polyomavirus Attenuates Cisplatin-Induced Apoptosis and Enhances E1, E6/E7, MMP-1, and Ki-67 Expression in HeLa Cervical Cancer Cells

**DOI:** 10.34172/apb.43882

**Published:** 2025-03-09

**Authors:** Fatemeh Pakdel, Seyed Masoud Hosseini, Neda Soleimani, Ali Farhadi

**Affiliations:** ^1^Department of Microbiology and Microbial Biotechnology, Faculty of Life Sciences and Biotechnology, Shahid Beheshti University, Tehran, Iran.; ^2^Division of Medical Biotechnology, Department of Medical Laboratory Sciences, School of Paramedical Sciences, Shiraz University of Medical Sciences, Shiraz, Iran.; ^3^Diagnostic Laboratory Sciences and Technology Research Center, School of Paramedical Sciences, Shiraz University of Medical Sciences, Shiraz, Iran.

**Keywords:** Cervical cancer, Cisplatin, sTAg, MCPyV, HPV-18, HeLa

## Abstract

**Purpose:**

Cervical cancer (CxCa) is primarily caused by high-risk human papillomaviruses (hrHPV), which disrupt p53 and pRb regulation, leading to uncontrolled growth and progression. Co-infection with polyomaviruses like MCPyV in some HPV-positive cases suggests a potential combined effect on tumor development. Cisplatin is commonly used for advanced CxCa, but resistance remains a challenge. This study examines whether MCPyV sT oncoprotein and HPV-18 oncoproteins affect key gene transcription, influencing proliferation and cisplatin resistance in CxCa.

**Methods:**

The sT gene was cloned into the pCMV6 vector, and HeLa cells were transfected with pCMV6-sT using Lipofectamine 3000. Transfection efficiency was assessed via fluorescence microscopy and flow cytometry. Protein expression was analyzed using SDS-PAGE and Western blotting. Cytotoxicity was measured with the MTT assay, gene expression was analyzed by RT-qPCR, Ki-67 staining was performed on cell blocks, and cisplatin-induced effects on proliferation and apoptosis were examined.

**Results:**

Cytotoxicity assays showed a significant increase in cell viability at 0.2 μg of sT plasmid after 72 hours (13.76%, *P*<0.05). MCPyV sT expression significantly upregulated E1 (4.22-fold), E6/E7 (3.80-fold), and MMP1 (6-fold) mRNA levels (*P*<0.001). Increased Ki-67 positivity indicated enhanced proliferation. Additionally, sT expression reduced cisplatin-induced apoptosis, with fewer apoptotic cells observed in the sT+cisplatin group than in the cisplatin-only group (25.9% vs. 38.3%, *P*<0.05).

**Conclusion:**

The presence of MCPyV sT and HPV oncoproteins together enhances resistance to cisplatin-induced apoptosis in CxCa cells, highlighting the need for further investigation into viral oncoprotein interactions to overcome therapeutic resistance.

## Introduction

 Cervical cancer (CxCa) is a widespread disease affecting women globally, causing significant morbidity and mortality.^1^ The primary cause of CxCa is infection with human papillomavirus (HPV), with over 200 identified types categorized by their oncogenic potential.^2^ Among the high-risk HPV types (hrHPV), HPV-18 is the second most prevalent, contributing to 65% of all cases of invasive CxCa worldwide.^2^ Infection with high-risk HPV types is known to contribute to the continued development and worsening of cervical dysplasia.^3^ HPV DNA integration into the host genome disrupts the E1/E2 regions, removing the E2 protein’s regulatory control over the E6 and E7 oncogenes.^4^ This leads to reduced p53 expression and degradation of both p53 and retinoblastoma (pRb) by E6 and E7, respectively, impairing cell cycle regulation and promoting uncontrolled cell proliferation, which can result in the development of high-grade cervical intraepithelial neoplasia (CIN) 2 and 3 lesions and invasive cancer.^3,5,6^ Furthermore, the E1 protein of HPV-16 and -18 can regulate genes involved in cell proliferation, migration, and metastasis, all of which contribute to cancer development.^7^

 While HPV infection is the primary cause of CxCa, its presence alone does not suffice for cancer development. The risk of cervical carcinogenesis may increase when other infections coexist with HPV.^8^ Co-infection with human polyomaviruses has been noted in tumors that test positive for hrHPV, indicating that these polyomaviruses could function as contributing factors in HPV-associated cancer development.^9^ Merkel cell polyomavirus (MCPyV), recognized as a causative agent of Merkel cell carcinoma (MCC), a highly aggressive form of skin cancer, has its DNA present in approximately 80% of MCC cases. Like hrHPV in CxCa, MCPyV genome integration has been identified in all virus-positive MCC cases studied.^10^ In cervical tumor samples, MCPyV DNA was found in 19% of hrHPV-positive cervical squamous cell carcinomas and 25% of hrHPV-positive cervical adenocarcinomas.^11^ Additionally, another study reported that 34% of hrHPV-positive cervical tumors were also MCPyV DNA positive.^10^

 The early region of the double-stranded DNA genome of MCPyV encodes the large T antigen (LT), small T antigen (sT), and 57 kT antigen (57kT).^12^ Both LT and sT inhibit growth suppression by interfering with pRb and p53, respectively.^13^ Integration often leads to tumor-specific truncation mutations that sustain the expression of sT and truncated large T antigen (LTT) mutants, which retain the critical N-terminal RB-binding LXCXE motif. This motif is essential for blocking the tumor-suppressing activity of pRb. LTT and sT, produced from the integrated viral genome, serve as the main oncoproteins promoting the ongoing growth and survival of tumor cells.^14^ While LT’s activation of p53 may have an anti-tumorigenic effect, sT’s inhibition of p53 tends to favor tumorigenesis.^15^ MCPyV sT can cause cellular transformation in Rat1 fibroblast cells and facilitate tumor development in p53-null transgenic mice, highlighting sT’s role as a major contributor to oncogenesis.^16,17^

 Ki-67 is a nuclear antigen found in all active phases of the cell cycle (G1, S, G2, M) except G0, and it indicates cell proliferation.^18^ Elevated Ki-67 expression signifies abnormal cell growth, particularly in advanced cervical lesions, and serves as a biomarker for diagnosing cervical lesions, providing insights into tumor progression, grading, and prognosis.^19,20^ Matrix metalloproteinases (MMPs), a group of zinc-containing endopeptidases, are essential for regulating tumor angiogenesis, invasion, and metastasis by remodeling and degrading the extracellular matrix.^21^ Dysregulation of MMP transcription, especially MMP1, can promote tumor metastasis.^22^ The overexpression of MMP1 is considered an unfavorable prognostic indicator in CxCa due to its role in degrading the extracellular matrix during tumor invasion.^23^

 Single-agent cisplatin (cis-diamminedichloroplatinum, CDDP) is the most effective treatment for advanced or recurrent CxCa.^24^ Its mechanism involves forming DNA adducts that initiate programmed cell death through key signaling pathways.^25^ CDDP-induced apoptosis is primarily regulated by the p53 protein, which interacts with Bcl-2 family proteins in the mitochondria and/or cytosol, activating the intrinsic apoptosis pathway. CDDP additionally triggers the activation of the ataxia telangiectasia and Rad3-related (ATR) kinase, resulting in phosphorylation and subsequent activation of p53.^26^ Despite CDDP’s effectiveness, many patients experience drug resistance upon relapse. Potential mechanisms contributing to cisplatin resistance include changes in cellular absorption and efflux, heightened metabolism and detoxification processes in the liver, as well as improved DNA repair and anti-apoptotic pathways.^27^ The E6 protein of HPV-16 has been shown to bind to p53, disrupting its transactivation and apoptotic functions, thereby contributing to platinum resistance.^26^

 Up to now, no research has examined whether the simultaneous expression of the sT oncoprotein of MCPyV, along with HPV-18 oncoproteins, affects the transcriptional regulation of key genes such as E1, E6/E7, and MMP-1, potentially promoting cell proliferation and reducing cisplatin-induced apoptosis in CxCa cells. This study aims to explore this relationship, which could provide new insights into the mechanisms of cell proliferation, metastasis, and cisplatin resistance in CxCa.

## Materials and Methods

###  Materials

 HeLa cells and *Escherichia coli* DH5α were sourced from the National Cell Bank of Iran (NCBI Code: C115, Pasteur Institute, Tehran, Iran). The following reagents were utilized: Dulbecco’s modified Eagle’s medium (DMEM), 10% fetal bovine serum (FBS), pfu polymerase, MTT solution, Dimethyl sulfoxide (DMSO), 3,3′-Diaminobenzidine (DAB), cisplatin, and 1% penicillin/streptomycin (all obtained from Sigma-Aldrich); trypsin solution (32778-05, Nacalai Tesque); EcoRI, XhoI, T4 DNA ligase, and Lipofectamine 3000 reagent (Thermo Fisher Scientific, Waltham, MA, USA); GFI Nucleic Acid extraction kit (Vivantis Technologies Co., Malaysia); polyvinylidene fluoride (PVDF) membranes (Millipore, Feltham, UK); RiboEx^TM^ total RNA extraction kit (GeneAll, Korea); PrimeScript RT Reagent Kit (TaKaRa, Japan); RealQ Plus 2x Master Mix Green (Ampliqon, Denmark); rabbit anti-human Ki-67 monoclonal antibody (Clone SP6, Vitro Master Diagnostica, Granada, Spain); EnVision + Dual Link System-HRP solution (Dako, Glostrup, Denmark); and PE-Annexin V Apoptosis Detection Kit (BD Biosciences, Bedford, MA, USA).

###  Cloning of the sT Gene into the pCMV6 Vector

 The sT gene, composed of 186 amino acids, was synthesized and first inserted into the pUC57 vector by BIOMATIK, based on the mRNA sequence from NCBI (GenBank ID: AEM01096.1). The gene was amplified using polymerase chain reaction (PCR) with pfu polymerase. Forward and reverse primers were designed using Gene Runner software (version 3.05, Hastings Software Inc., Hastings, NY, USA) with EcoRI and XhoI restriction sites at the N- and C-termini, respectively. The primer sequences were: Forward: 5′-GAATTCATGGATTTAGTCCTAAATAG-3′, and reverse: 5′-CTCGAGCTAGAAAAGGTGCAGAT-3′. The resulting amplification fragment of the sT gene was inserted into the pCMV6-AC-IRES-GFP mammalian expression vector using T4 DNA ligase. Competent *E. coli* DH5α cells were transformed with the recombinant plasmid containing the sT gene (pCMV6-sT). The vector was authenticated through colony PCR, enzyme digestion, and Sanger sequencing. The Sanger sequencing results were analyzed using FinchTV software (version 1.4.0; http://www.geospiza.com/product/finchtv.shtml). Subsequently, the recombinant plasmids were amplified in *E. coli* DH5α and purified using the GFI Nucleic Acid extraction kit, according to the manufacturer’s instructions.

###  Cell culture and vector transfection

 HeLa cells were cultured in a complete growth medium (DMEM, 10% FBS, and 1% penicillin/streptomycin) and incubated at 37 °C in a 5% CO2 environment. After 24 hours of growth, cells were transfected with pCMV6-sT and mock control plasmids. For transfection, cells were seeded in 6-well plates at a density of 8 × 10^5^ cells per well. Upon reaching 60%–70% confluence, cells were transfected using Lipofectamine 3000 reagent, following the manufacturer’s protocol. The Lipofectamine 3000 reagent and DNA were mixed, and the complex was incubated at room temperature for 15 minutes before being introduced to the cells. After transfection, cells were washed with PBS and cultured in fresh media for 4-6 hours.

###  Fluorescence microscopy and flow cytometry analysis

 Transfection efficiency was assessed using both transmission and fluorescence microscopy. Transfected cells were examined under an Olympus microscope with a 20x objective lens at 24, 48, and 72 hours post-transfection. Fluorescent images were captured using a GFP filter cube, and transfection efficiency was quantified by counting green and non-green cells. Four images were taken at each time point, with 100 cells analyzed per image. For flow cytometry, cells were washed with PBS at 48 and 72 hours post-transfection, detached with trypsin-EDTA, and resuspended in PBS after centrifugation (350 × g for 5 minutes). Fluorescent intensity was evaluated using a FACSCalibur flow cytometer (BD Biosciences, California, USA), analyzing 10 000 cells per experiment. Data were processed with FlowJo software (version 10.0).

###  SDS-PAGE and western blotting

 Cell lysates were prepared as previously described and mixed with 5 × sample buffer, then boiled at 95 °C for 10 minutes.^28^ Samples were subjected to SDS-PAGE using the Laemmli method.^29^ Equal protein amounts from each sample were electrophoresed on 12% SDS-polyacrylamide gels and transferred onto PVDF membranes using a Bio-Rad Mini Trans-Blot® electrophoretic transfer cell (300 mA for 3 hours). Membranes were incubated in a blocking solution (5% skim milk, 0.05% Tween 20) for 16 hours, then washed with TBST (150 mM NaCl, 50 mM Tris-base, 0.05% Tween 20) for 15 minutes. sT protein abundance was measured using a polyclonal anti-sT antibody raised in rabbits through standard immunization protocols.^30^ The rabbit was initially immunized with 500 μg of synthetic peptide and boosted twice with 250 μg of the same antigen at 3-week intervals. The peptide MDLVLNRKERREALC, corresponding to MCV small T antigen exon 1 as described by Kwun et al,^31^ was synthesized by Proteogenix (Oberhausbergen, France) with > 95% purity. An anti-β-actin antibody (diluted 1:5000, Santa Cruz Biotechnology) served as an internal control during sT protein immunoblotting. After three washes with TBST, a horseradish peroxidase-conjugated secondary antibody (1:10 000, Sigma-Aldrich) was applied to the membrane and incubated for 1 hour at room temperature, followed by two washes with TBS buffer (50 mM Tris-base, 150 mM NaCl). Finally, a substrate solution containing 5 mg of DAB and 5 µL of H₂O₂ in 10 mL of ddH₂O was added to initiate the reaction, which was terminated by adding ddH₂O.

###  Cytotoxicity assay

 The cytotoxicity of the sT protein was assessed using the MTT assay. HeLa cells were seeded in 96-well plates at a density of 8000 cells per well in 200 μL of culture medium and incubated at 37 °C with 5% CO2. Upon reaching 70% confluence, cells were transfected with varying amounts of pCMV6-sT and mock plasmid (0.1 to 0.25 μg). Cell viability was evaluated at 24, 48, and 72 hours post-transfection by adding fresh medium containing 20 μL of MTT solution (5 mg/mL) to each well and incubating for 4 hours. Afterward, 150 μL of DMSO was added to each well, and absorbance was measured at 570 nm using a Sat Fax 2100 microplate reader (Stat Fax, USA). The experiment was conducted in triplicate.

###  Reverse transcription quantitative PCR analysis

 To analyze small T antigen mRNA expression levels, HeLa cells transfected with pCMV6-sT and mock plasmid were collected at 48 and 72 hours post-transfection. Total RNA was extracted using the RiboEx^TM^ kit, and cDNA was synthesized at 37 °C for 15 minutes with the PrimeScript RT Reagent Kit. DNase was used to eliminate potential plasmid contamination. The experiment was conducted using the ABI Prism 7000 Sequence Detection System (Applied Biosystems) and RealQ Plus 2x Master Mix Green. Primer sequences for RT-qPCR were designed using Gene Runner software and are detailed in Table 1. Standard curves were established to assess primer efficiency, and the relative fold changes of target genes were calculated following the Pfaffl method,^32^ with glyceraldehyde-3-phosphate dehydrogenase (GAPDH) as the housekeeping gene (Forward: GGCCTCCAAGGAGTAAGACC, Reverse: AGGGGTCTACATGGCAACTG).^33^ The thermocycling protocol included initial denaturation at 95˚C for 5 minutes, followed by 40 cycles of 95 ˚C for 15 seconds, annealing at a specific temperature for 20 seconds, and extension at 72 ˚C for 25 seconds.

**Table 1:** Oligonucleotide primer sequences used for reverse-transcription real-time PCR assay

**Gene**	**Sequence (5ʹ to 3ʹ)**	**PCR product size (bp)**	**Annealing temperature (˚C)**
sT-Forward	GGAATTGAACACCCTTTGG	236	51
sT-Reverse	CTACAATGCTGGCGAGACA		
E1-Forward	CATTTACCAGCCCGACGA	236	55
E1-Reverse	AAACCAGCCGTTACAACCC		
E6/E7-Forward	GACATTGTATTGCATTTAGAGCC	155	56
E6/E7-Reverse	CATTGTGTGACGTTGTGGTTC		
MMP1-Forward	TGAAAAGCGGAGAAATAGTG	153	55
MMP1-Reverse	GAGGACAAACTGAGCCACAT		

###  Ki-67 Immunocytochemistry

 To assess the impact of the MCPyV sT protein on the proliferation of HeLa cells, Ki-67, a well-established proliferation marker, was employed to evaluate the growth fraction of the cell populations.

####  Cell block preparation

 HeLa cells were transfected with either pCMV6-sT or a mock plasmid, along with untreated cells. After collection, the cells were washed twice with cold PBS and centrifuged at 300 × g for 10 minutes. Cell blocks were prepared following the plasma-thrombin method described by Poojan et al.^34^ The resulting clot was transferred to a tissue bag and cassette, fixed in formalin, and processed in a Shandon Citadel 2000 tissue processor. Serial sections (3-4 μm) were cut from the cell blocks, deparaffinized in xylene, and underwent epitope retrieval via heat-induced microwave treatment in 10 mM citrate buffer (pH 6.0). Endogenous peroxidase activity was inhibited using 10% H₂O₂, and non-specific binding sites were blocked with a protein blocking agent (Dako, Glostrup, Denmark). The slides were incubated for 1 hour at room temperature with a 1:200 dilution of rabbit anti-human Ki-67 monoclonal antibody in a humidified chamber, followed by a 30-minute incubation with EnVision + Dual Link System-HRP solution. DAB served as the chromogen in the presence of hydrogen peroxide, while nuclei were counterstained with Meyer’s hematoxylin. Slides without primary antibody acted as negative controls.

####  Interpretation of Ki-67 immunostaining findings

 Ki-67 staining was scored semi-quantitatively based on labeling intensity (0 = absent; 1 = weak; 2 = moderate; 3 = strong) and the percentage of positive cells (0%–100%). A final score was calculated by multiplying the percentage of positive cells by the intensity, with a maximum possible score of 300.^35^ Two independent investigators assessed the scores, with inter-observer differences of less than 4%, and the average of their values was used.

###  Treatment of cells with CDDP and cell proliferation assay

 HeLa cells were seeded into 96-well plates at a density of 8000 cells per well and incubated at 37 °C with 5% CO₂. Upon reaching 70% confluence, the cells were transfected with either pCMV6-sT or mock control plasmids, following the transfection protocol from Thermo Fisher Scientific. After a 24-hour incubation to allow adherence and to reach 50%–60% confluence, the cells were treated with varying concentrations of cisplatin (1 to 4 μg/mL) based on its previously determined inhibitory concentration (IC50).^36^ Cell proliferation was evaluated 72 hours post-transfection. Control groups included cells treated with CDDP and untreated cells that were neither transfected with pCMV6-sT nor the mock plasmid.

###  Apoptosis assay

 The level of apoptosis was assessed in 5 × 10^5^ transfected HeLa cells. After 72 hours, the cells were washed with 1 mL of PBS and detached using 250 μL of trypsin solution, followed by centrifugation at 350 × g for 5 minutes. The supernatant was discarded, and the cells were washed twice with cold PBS. The apoptosis assay was conducted using a PE-Annexin V Apoptosis Detection Kit according to the manufacturer’s instructions. In brief, HeLa cells transfected with pCMV6-sT, mock plasmid, and untreated cells were resuspended in 400 μL of binding buffer, incubated with 5 μL of PE-conjugated Annexin V in the dark for 15 minutes, and subsequently stained with 7-AAD. After an additional 15-minute incubation at room temperature, flow cytometric analysis was performed using a FACSCalibur flow cytometer (BD Biosciences, San Jose, CA, USA). Data analysis was conducted with FlowJo software version 10.0.

###  Statistical analysis

 Statistical analyses were performed with GraphPad Prism software version 8.0 (GraphPad Software Inc., La Jolla, CA, USA). Given the non-normal distribution of the data, we applied the Kruskal-Wallis test, a non-parametric approach, to compare the mean differences across multiple independent groups and determine statistical significance. Results are expressed as means ± SD, with a p-value of less than 0.05 considered statistically significant.

## Results and Discussion

###  Evaluation of pCMV6-sT-GFP cloning, transfection, and MCPyV sT protein expression in HeLa cells

 To verify the successful insertion of the sT gene into the pCMV6-AC-IRES-GFP vector, PCR amplification was performed. Agarose gel electrophoresis displayed a 571-bp band, confirming the cloning of the sT gene. Dual digestion of the recombinant pCMV6-sT plasmid yielded bands at approximately 571 bp and 6729 bp, providing evidence of accurate cloning. Further validation was achieved through Sanger sequencing, which confirmed the fidelity of the cloning process.

**Figure 1:**
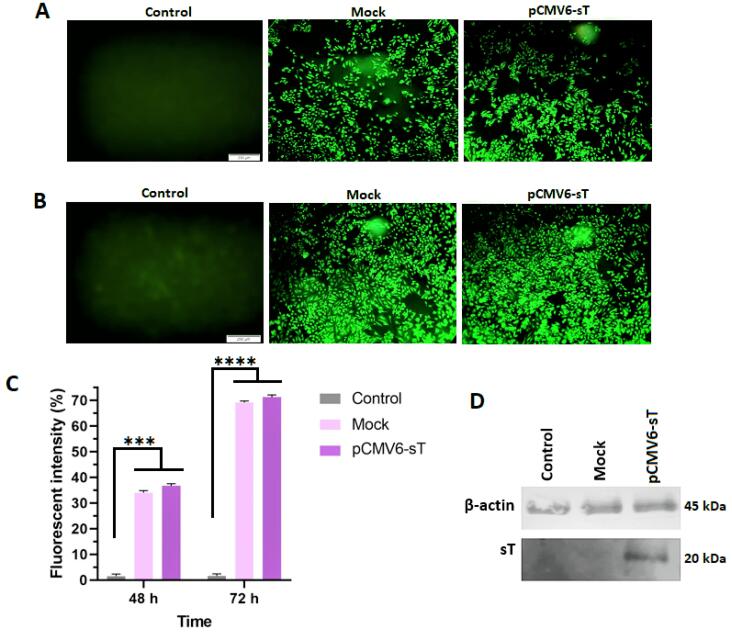
Fluorescent intensity measurement for pCMV6-sT, mock-transfected, and control groups using a fluorescence microscope and flow cytometer. The results from the fluorescence microscope showed that the percentage of transfected cells with pCMV6-sT and pCMV6 exceeded 35% at 48 hours (A) and 70% at 72 hours (B) post-transfection in HeLa cells. Flow cytometry analysis revealed that the average fluorescent intensity of pCMV6-sT and mock-transfected cells significantly increased after 48 hours (*P* ≤ 0.001) and 72 hours (*P* ≤ 0.0001) compared to the control cells (C). The sT protein level in HeLa cells was determined by Western blotting (D), which showed the expression of sT in HeLa cells transfected with the pCMV6-sT plasmid compared to the untreated and mock groups. ***P < 0.001, and ****P ≤ 0.0001

 Transfection efficiency was evaluated using fluorescence microscopy, which revealed that over 70% of HeLa cells were transfected with either the mock or pCMV6-sT plasmids (Figure 1A and B). Flow cytometry, conducted at 48 and 72 hours post-transfection, showed efficiencies of 36.7% and 71.3%, respectively, for pCMV6-sT (Figure 1C).

 Western blot analysis confirmed high levels of MCPyV sT protein expression in pCMV6-sT-transfected HeLa cells, with no detectable expression in untreated or mock-transfected cells (Figure 1D).

###  Cytotoxicity assay

**Figure 2:**
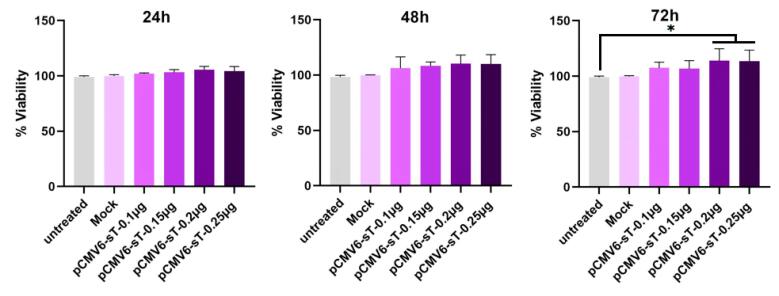
HeLa cells were transfected with the pCMV6-sT plasmid (ranging from 0.1 to 0.25 µg) and a mock plasmid (0.2 µg), and /their viability was evaluated by MTT assay after 24, 48, and 72 hours. The MTT assay revealed that cell viability increased by 5.42%, 10.35%, and 13.76% at a concentration of 0.2 µg of pCMV6-sT after 24, 48, and 72 hours, respectively, compared to untreated and mock-transfected cells. Data are presented as mean ± SD from three independent experiments. *P < 0.05

 HeLa cells were treated with varying concentrations of pCMV6-sT and mock plasmids (0.1, 0.15, 0.2, and 0.25 μg of DNA) for 24, 48, and 72 hours. After 72 hours, transfection with 0.2 μg of pCMV6-sT led to a significant 13.76% increase in cell viability relative to untreated and mock-transfected cells (*P <*0.05). No significant changes in viability were observed at 24 or 48 hours (P > 0.05) (Figure 2).

###  Effect of MCPyV sT protein expression on E1, E6/E7, and MMP1

**Figure 3:**
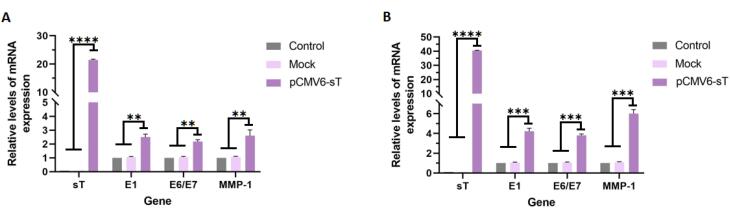
HeLa cells were transfected with pCMV6-sT and mock plasmids for 48 and 72 hours. The mRNA expression levels of sT, E1, E6/E7, and MMP1 genes were determined by RT-qPCR at 48 hours (A) and 72 hours (B) post-transfection. The mRNA expression of the sT gene was significantly higher in HeLa cells transfected with the pCMV6-sT plasmid compared to the mock and control groups (*P* ≤ 0.0001). pCMV6-sT transfection led to an increase in E1, E6/E7, and MMP1 gene expression at 48 hours (*P* < 0.005) and 72 hours (*P* < 0.001) post-transfection, compared to the mock and control groups in HPV-18-infected HeLa cells. **P < 0.01, ***P < 0.001, and ****P ≤ 0.0001

 The effects of HPV E6 and E7 on cell cycle disruption, apoptosis inhibition, and genomic instability are well-known, though E1’s role beyond viral replication remains unclear. E1 has been found to drive host cell proliferation and interact with other host proteins, emphasizing its emerging role in cervical carcinogenesis.^37^ In this study, we assessed the expression of E1 and E6/E7 in untreated, mock-transfected, and pCMV6-sT-transfected cells. RT-qPCR analysis showed that MCPyV sT expression significantly increased E1 and E6/E7 mRNA levels in pCMV6-sT-transfected HeLa cells compared to controls, with *P <*0.005 at 48 hours and *P <*0.001 at 72 hours (Figures 3A and 3B).

 A previous study has shown that MCPyV full-length LT, sT, or a combination of sT and LT enhances the transcriptional activity of HPV-18 LCR in C33A and HSC-3 cells, with cell-type-specific effects.^10^ Research indicates that the LT and sT proteins of the polyomavirus SV40 stimulate HPV promoters in various cell lines, suggesting that MCPyV sT may play a similar role. Specifically, SV40 LT increased HPV-18 promoter activity 13-fold in HeLa cells and approximately threefold in both 3T6 and SW13 cells.^38^ In human keratinocytes, the presence of both LT and sT led to a nine-fold increase in HPV-18 promoter activity.^39^ In human embryonic fibroblasts, SV40 sT stimulated HPV-16 promoter activity by 20- to 30-fold, whereas LT’s effect was 5- to 6-fold weaker.^40^ In line with these findings, the present study showed that MCPyV sT increased E1 and E6/E7 mRNA levels of HPV-18 by 4.22- and 3.80-fold, respectively. The mechanism by which MCPyV sT enhances HPV-18 LCR activity remains unclear, though it may not involve the typical sT interactions with HSC70, PP2A, or Fbxw7, as described by Rasheed et al.^10^ Further research is needed to identify the specific LCR sequences responsible for sT-induced activation.

 Studies have shown that the elevated MMP1 levels are linked to poor CxCa prognosis.^41^ Monitoring MMP1 expression could help predict the aggressiveness of the disease and potential outcomes.^42^ We assessed MMP1 mRNA expression levels in untreated, mock-transfected, and pCMV6-sT-transfected groups. The results demonstrated that MCPyV sT expression in HeLa cells transfected with the pCMV6-sT plasmid led to a significant 6-fold increase in MMP1 gene expression at the transcriptional level, compared to both the control and mock-transfected groups (*P <*0.001), as shown in Figures 3A and 3B. Taken together, the elevated levels of E1, E6/E7, and MMP1 resulted in an increased proliferation index in HeLa cells. This observation will be discussed in more detail in the following section.

###  MCPyV sT protein increases proliferation in HeLa cells

**Figure 4:**
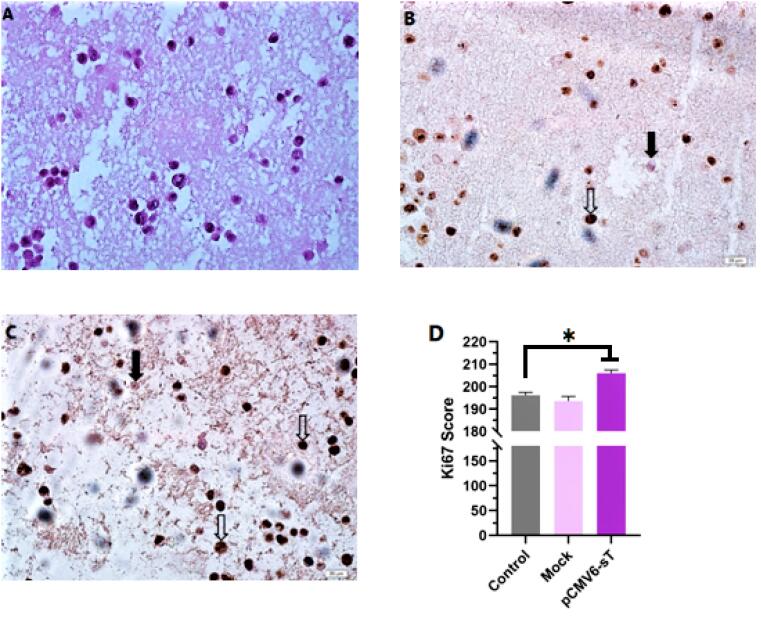
Immunocytochemistry analysis of Ki-67 expression in formalin-fixed paraffin-embedded (FFPE) HeLa cell blocks. (A) Hematoxylin and eosin stained image of HeLa cells in a FFPE section. (B) Untreated Hela cells. (C) HeLa cells 72 hours after treatment with pCMV6-sT. Hollow arrows and solid arrows indicate cells that are positive and negative for the Ki-67 marker, respectively. Scale bar = 20 µm. (D) Post-treatment with pCMV6-sT for 72 hours increases Ki-67 expression. Results are presented as the mean ± SD of three independent experiments, each performed in triplicate. *P < 0.05

 Ki-67 is widely acknowledged as an important prognostic biomarker in cervical carcinoma research, with its expression typically being highest in tumors exhibiting poor clinical and histopathological characteristics.^43^ This marker is detected throughout the active phases of the cell cycle, including G1, S, G2, and M, but is absent in the dormant G0 phase.^44^ In the present study, immunocytochemistry results confirmed the expression of Ki-67 in the cell nuclei of pCMV6-sT-transfected, mock-transfected, and control cells. While most of the HeLa cell nuclei were Ki-67 positive, as anticipated (Figures 4A and 4B), a notable difference in the Ki-67 expression score was observed after 72 hours between pCMV6-sT-transfected HeLa cells and mock-transfected or untreated cells (*P <*0.05). Importantly, Ki-67 expression was observed in both untreated and mock-transfected cells; however, the proportion of Ki-67 expression was markedly elevated in HeLa cells transfected with pCMV6-sT. The immunohistochemistry results showed only a few brown cells in the untreated cells and mock-transfected cells. These results indicate that the MCPyV sT protein significantly enhances Ki-67 expression, suggesting increased proliferation in HPV-18-infected HeLa cells (Figure 4C). Although a high Ki-67 index is often associated with the severity of cervical lesions and is typically not correlated with HPV infection,^43^ Ki-67 can still be expressed during cell proliferation triggered by HPV infection and may progress with the presence of the MCPyV sT protein. Thus, enhanced immunocytochemical staining of pCMV6-sT-transfected cells with Ki-67 validated the proliferative impact of the MCPyV sT protein in HeLa cells.

 The activation of noncanonical NF-κB (ncNF-κB) signaling by MCPyV sT and its interaction with the cellular phosphatase PP2A and the TIP60 complex, which are involved in various signaling pathways, including those regulating the cell cycle and proliferation, is crucial for promoting tumorigenesis and enhancing cellular proliferation.^45,46^ Consistent with our findings, it has been shown that the activation of these pathways by MCPyV sT correlates with enhanced cell proliferation in MCC.^45,47^ Conversely, while MCPyV sT promotes proliferation, the potential for therapeutic interventions targeting these pathways remains an area of active research, suggesting that inhibiting sT or its downstream effects could provide new strategies for cancer treatment.

###  MCPyV sT protein inhibits cisplatin-induced cytotoxicity

**Figure 5:**
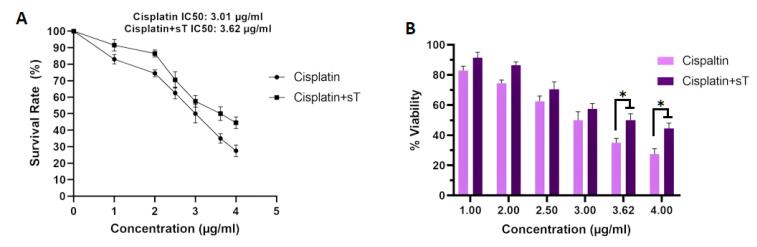
(A) Representative survival curve of cells treated with cisplatin alone and in combination with pCMV6-sT after 72 hours. The IC50 value of cisplatin (CDDP) increased from 3.01 to 3.62 μg/mL following transfection with pCMV6-sT and combination treatment. (B) HeLa cells were treated with cisplatin alone and in combination with pCMV6-sT for 72 hours, and cell viability was assessed by MTT assay. Data are presented as the mean ± SD of three independent experiments. *P < 0.05

 Cell viability was evaluated using the MTT assay following transfection of HeLa cells with pCMV6-sT, treatment with cisplatin, and a combination of both. Our findings indicated that treatment with cisplatin alone resulted in approximately 50% cell survival at a concentration of 3.01 μg/mL compared to the control group. Subsequently, cells were treated with pCMV6-sT combined with cisplatin (sT + cisplatin [1-4 μg/mL]) for 72 hours. The results from the MTT assay (Figures 5A and 5B) demonstrated that MCPyV sT protein increased the IC50 value of cisplatin to 3.62 μg/mL, suggesting that sT protein reduces the effective dose concentration of the drug. Furthermore, when the cells were treated with a combination of cisplatin and pCMV6-sT, the viability rate increased from 35.02% to 50.11% after 72 hours, indicating a statistically significant difference (*P <*0.05). Earlier research has shown that cisplatin reduces the expression of E6/E7 oncogenes in HPV-infected tumor cells,^48^ preventing E6 from degrading TP53 and resulting in the accumulation of TP53 within the nucleoli of HeLa cells.^49^ Consequently, it has been suggested that combining cisplatin with siRNA targeting E6, E6/E7, or HPV-16 E6/E7-CRISPR/Cas9 could synergistically restore the function of TP53 and/or pRB, which may provide a more effective treatment for CxCa compared to using either strategy individually.^50,51^ On the other hand, cytotoxic chemotherapy (cisplatin combined with etoposide), which is commonly used to treat patients with advanced MCC, often results in limited durability of response, and only a few studies have demonstrated a survival benefit.^52,53^ Our results indicate that the transfection of MCPyV sT oncoprotein promotes HeLa cell proliferation in the presence of cisplatin compared to controls. MCPyV sTAg facilitates anchorage-independent growth, serum-independent growth, and loss of contact inhibition, all of which are crucial for MCC tumor cell proliferation.^16^ Additionally, in vitro studies have shown that MCPyV sTAg, rather than LTAg, drives a transformed phenotype in rodent fibroblasts by inhibiting the cellular ligase SCFFbw7, resulting in the accumulation of oncoproteins such as cyclin E and c-Myc.^16,54^ Hence, it can be concluded that MCPyV sTAg enhances the ability of HeLa cells to proliferate and grow, even in the presence of cisplatin.

###  MCPyV sT protein inhibits cisplatin-induced apoptosis

**Figure 6:**
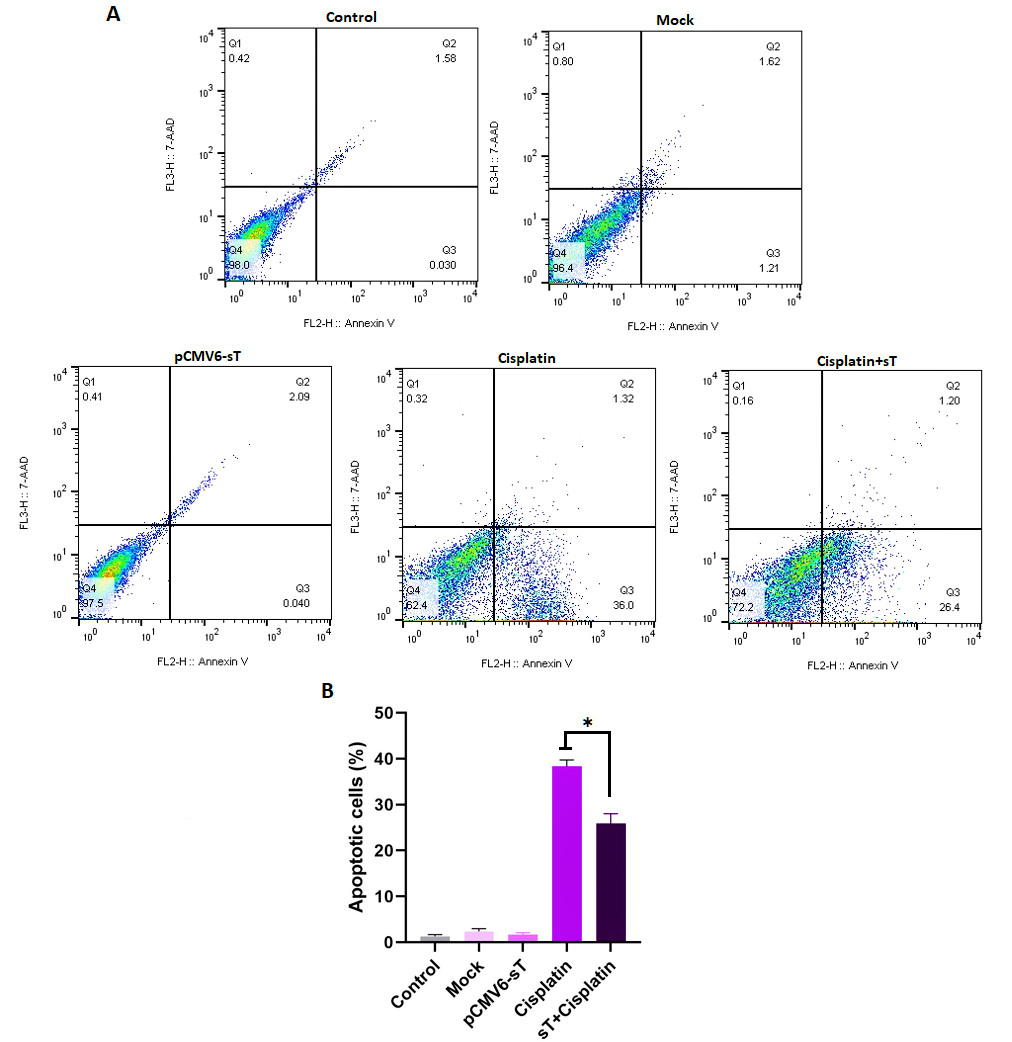
The effect of MCPyV sT protein on cisplatin-induced apoptosis in HeLa cells. FlowJo software version 10.0 was utilized to generate the flow cytometry plots. The histogram represents the percentage of total apoptotic cells in different groups (A). A statistically significant decrease in the number of apoptotic HeLa cells was observed in the cisplatin + pCMV6-sT group compared to the cisplatin-alone group (*P* < 0.05). However, the difference in the number of apoptotic cells between the untreated, pCMV6-sT, and mock groups was not significant (*P* > 0.05) (B). Q1, Q2, Q3, and Q4 indicate necrotic, late apoptotic, early apoptotic, and viable cells, respectively. The percentage of total apoptotic cells was calculated from Q2 + Q3. *P < 0.05

 Cisplatin is a vital medication for treating CxCa due to its capacity to inhibit tumor cell growth by causing DNA damage and triggering apoptosis.^55^ The stability and activation of wild-type p53 are crucial for the apoptotic process induced by cisplatin. When p53 is absent, apoptosis is hindered, resulting in a tolerance to DNA damage and subsequent drug resistance.^56^ The detection of MCPyV DNA in cervical tumor samples^57,58^ has raised concerns that the small T oncoprotein of MCPyV, along with HPV-18 oncoproteins, may reduce the effectiveness of cell death induced by cisplatin in CxCa cells. In this study, the anti-apoptotic effects of MCPyV sT protein on cancer cells were examined through flow cytometry analysis using Annexin V/PI, as illustrated in Figure 6. HeLa cells treated with cisplatin alone showed a significant increase in apoptotic cell death, reaching 38.3% after 72 hours, which was substantially higher than that observed in the mock and untransfected pCMV6-sT groups (*P <*0.001). However, in cells transfected with the pCMV6-sT vector before cisplatin treatment, the percentage of apoptotic cells (25.9%) markedly reduced in comparison to the cisplatin-only group (*P <*0.05).

 While some studies suggest that MCV LT or sT oncoproteins do not have a p53 targeting domain,^59,60^ recent research indicates that MCPyV sT induces transcriptional changes that upregulate at least two genes: MDM2, which facilitates the proteasomal degradation of p53, and lysine-specific histone demethylase 1A (LSD1), which is crucial for preserving the plasticity and proliferative potential of MCC cells.^61,62^ This process is largely driven by the upregulation of MCT-1, which antagonizes p53 function.^63^ As a result, p53 levels are reduced, impairing its ability to initiate apoptosis and promoting cell survival in oncogenic contexts. The loss of functional p53 due to degradation contributes to resistance to apoptosis, allowing cells to survive under genotoxic stress.^64^ Additionally, the interaction between p53 and miR-191-5p further amplifies survival signals, further complicating apoptosis resistance.^65^ MCPyV sT also influences these transcriptional alterations through the recruitment of a MYCL/MAX heterodimer to the EP400 complex.^66^ Although MCPyV sT does not directly target p53 like HPV E6 protein, it still promotes p53 degradation by modulating downstream genes, representing a unique mechanism by which MCPyV sT disrupts apoptosis regulation.

 Notably, Shuda et al found that MCPyV sT enhances the proliferation of Merkel cell progenitors in embryonic mice and can fully transform cancer cells in a p53-null environment.^17^ They showed that prolonged MCPyV sT expression in p53-null transgenic mice, mimicking the p53 status in HPV-infected CxCa cells, leads to poorly differentiated neoplasms in the spleen and liver, underscoring the importance of the p53 tumor suppressor pathway in sT-induced tumorigenesis.^17^ This suggests that MCPyV sT may protect CxCa cells from cisplatin-induced apoptosis highlighting the need for novel therapeutic strategies to overcome this resistance. The combined expression of MCPyV sT and hrHPV oncoproteins may impact apoptosis pathways by modulating key signaling mechanisms. HPV E6 and E7 activate the PI3K/Akt pathway, which is crucial for cell survival and proliferation,^67^ and inhibit apoptosis by targeting pro-apoptotic proteins like p53 and FADD via the ubiquitin-proteasome pathway.^68^ Additionally, HPV E5 can impair extrinsic apoptosis pathways, further promoting cell survival.^67^ In parallel, MCPyV sT enhances the transcriptional activity of HPV oncoproteins, which may amplify their anti-apoptotic effects.^10^ The interaction between MCPyV and HPV oncoproteins may also lead to altered expression of cellular microRNAs, further disrupting apoptosis signaling.^67^ This reciprocal activation suggests a synergistic relationship that could enhance oncogenic potential.

 Targeting key pathways involved in apoptosis regulation, particularly the p53 pathway, could restore cisplatin sensitivity. Specific inhibitors that disrupt the interaction between MCPyV sT, HPV oncoproteins, and p53 or small molecules and RNA-based therapies that specifically target the viral oncoproteins to restore p53 function, may enhance chemotherapy effectiveness. Additionally, combining cisplatin with agents that reverse resistance mechanisms, such as NF-κB inhibitors, could improve therapeutic outcomes. Immunotherapies, including immune checkpoint inhibitors, could also restore immune surveillance and sensitize tumor cells to cisplatin. Given the complexity of viral oncoprotein interactions, combination therapies targeting multiple pathways may provide a promising approach to improving clinical responses in HPV-associated cancers resistant to chemotherapy. Investigating these interactions could uncover new therapeutic targets and strategies to improve the efficacy of cisplatin and other chemotherapy agents in treating virus-associated cancers.

## Conclusion

 In summary, our study demonstrates that the simultaneous presence of MCPyV sT and HPV oncoproteins enhances resistance to cisplatin-induced apoptosis, particularly in HPV-18-infected CxCa cells. These findings suggest that MCPyV sT alters the expression of genes associated with p53 degradation, contributing to a cellular environment less responsive to cisplatin-induced apoptosis. Clinically, this emphasizes the complexity of viral co-infections in cancer cells and their potential role in chemotherapy resistance, which may complicate treatment outcomes in patients with virus-associated cancers. However, the study’s limitations, such as its focus on in vitro models, warrant caution in directly extrapolating these results to clinical settings. Future research into the molecular mechanisms underlying this interaction, as well as the validation of these findings in clinical samples, could inform the development of novel therapeutic strategies to overcome chemotherapy resistance in virus-associated cancers.

## Competing Interests

 The authors declare no conflicts of interest.

## Data Availability Statement

 All relevant data are within the manuscript, and any other additional materials are available upon request.

## Ethical Approval

 In this study, all the experiments were approved by the Institutional Ethics Committee (approval number: (IR.SBU.REC.1403.050), Shahid Beheshti University, Tehran, Iran.

## Informed Consent Statement

 Not applicable.

## Institutional Review Board Statement

 The Institutional Review Board and the Research Ethics Committee at Shahid Beheshti University approved the study (Approval Number: IR.SBU.REC.1403.050, dated 2024-05-11).
